# Model organisms in Ayurvedic research

**DOI:** 10.4103/0975-9476.72610

**Published:** 2010

**Authors:** Madan Thangavelu

**Affiliations:** *26 Stansgate Avenue, Cambridge, CB2 0QZ, UK E-mail: madan.thangavelu@gmail.com*

Sir,

Ayurveda states that *jara* (aging) is a “*swabhavika”* (natural) property of all organisms – unicellular and multicellular. Interventions ranging from environmental, life style, genetic, and sometimes even chemical to delay *kalaja jara* (predictable and unavoidable aging) and prevent *akalaja jara* (untimely or avoidable aging) should be and are possible. Of the examples now available, some use as little as a single, simple chemical. In 2002, for instance, the late Seymour Benzer and colleagues reported life extension in Drosophila[1] by feeding a “drug” 4-phenylbutyrate (PBA). The studies also provided early evidence of the mode of action… “a global increase in histone acetylation as well as a dramatically altered pattern of gene expression, including induction or repression of numerous genes.” Aging and longevity are clearly very complex and real traits.

Such studies seem to support Ayurveda’s “*Rasayana Tantra,”* which claims innumerable rejuvenating and life-extending practices and formulations (see Prof. Ajay Kumar Sharma’s “Elements of Rasayana Therapy in Ayurveda,” ISBN 81-7030-831-3, for a brief but detailed account of Rasayanas). The paper by Priyadarshini *et al*. [*J-AIM* 1(2): 114–119] shows that similar studies can be performed using rasayanas, but raises questions about this important but underexplored and underappreciated area. The most significant aspects of the experiments and their results as reported in the paper are their obvious weakness! By not providing details of either the formulation or the way in which it was arrived at, and by doing so in the manner done… the paper should initiate an important debate.

Such debates hold latent benefits – I illustrate this using an example from a similar set of circumstances, with which I was closely involved in the early days of plant DNA research. In the late 1970s and the early 1980s, the big challenge was being able to prepare high-quality plant DNA from a tissue rich in phenolics and other secondary compounds, which readily complexed with the DNA making it unsuitable for any kind of enzymatic investigation. Such was the order of magnitude of this problem that several meetings were held around Europe with the sole aim of bringing together researchers to discuss methods to improve results. Later, in the mid-1980s, several informal and very short contributions helped shape and speed the generation of plant DNA libraries in bacteriophage lambda cloning vectors purely by appropriate selection of bacterial hosts. These important and nontrivial successes were due to researcher skills based on deep appreciation of bacterial genetics.

The single example below from the Maize Genetics Cooperation Newsletter (http://www.agron.missouri.edu) was particularly significant and is worth citing as its insights and choice of bacterial strain helped very rapidly move the entire field of construction of plant genomic DNA libraries in bacteriophage lambda cloning vectors, and enabled the isolation and characterization of genes from several plant species – all to be carried out single-handedly by a PhD student like me! The background to the challenges faced in choice of bacterial host strains like *Escherichia coli* Q358 and K803 and their genotypes and compatibility with Enterobacteria phage λ (lambda phage)-based cloning vectors available are all in this brief contribution by Nina Federoff. I quote below (in italics) from:Notes on cloning maize DNA by Nina Fedoroff Maize Genetics Cooperation Newsletter; Volume 57, 1983 [http://www.agron.missouri.edu/mnl/57/125fedoroff.html]

To determine whether the inability to form plaques on Q358 is an inherent property of recombinant phage containing maize DNA, recombinant phage were propagated on K803 and tested for their ability to grow on the various strains. The results are shown in Table 2 and indicate that once the maize DNA has been “laundered” through E. coli, the recombinant phage grow equally well on Q358, Q364 and K803. Since plant DNA’s are known to be more extensively modified than other DNA’s, it appears a reasonable conjecture that the difference in plating efficiency is attributable to differences among E. coli strains in the ability to replicate heavily modified DNAs.

Subtle experimental “innovations” of such kinds have great significance, which most scientists do not realize, and details of which are often forgotten. The pioneers of those early plant DNA meetings and discussions went on to play leading roles in all subsequent plant DNA research. Nina Vsevolod Fedoroff (born 1942), the author of this small piece in the *Maize Genetics Cooperation Newsletter*, is now Willaman Professor of Life Sciences and Evan Pugh Professor of Biology, Penn State University, and was the founding director of the Huck Institutes of the Life Sciences, a life sciences consortium. She went on to receive the 2006 National Medal of Science in the field of biological sciences, and is currently Science and Technology Adviser to U.S. Secretary of State Hillary Clinton (http://en.wikipedia.org/wiki/Nina_Fedoroff).

The missing details about the development of “Drosophila-specific Rasayanas” in the paper by Priyadarshini *et al*, raise the need for debates and other events similar to those in the early days of plant DNA research. Open discussions and interactions between researchers based on deep understanding of Ayurvedic basic principles and the strengths and powers (and limitations) of model organisms should encourage all to consider the fine details – after all, both “God” and the “Devil” are hidden in details!

The field is of importance, because many concepts fundamental to Ayurveda’s “Rasayana Tantra” should be testable in suitable *D. melanogaster* models, particularly cellular and subcellular mechanisms of action of rasayanas’ chemical aspects. This is similar to the way that concepts in western bioscience are presently tested in them. Some might even be directly applicable to vertebrates and higher model organisms and might show ways to benefit humans. The “drug” used by Benzer’s team, 4-PBA, is now known to be a histone deacetylase inhibitor, and could well provide a way to prevent cataract[2] and fibrotic disorders[3]

It may be that only experimenters like me will appreciate the significance of such small details, but losing the opportunity presented by the incompleteness in Priyadarshani *et al*, could be tragic for future Ayurveda research of this kind. Might I suggest holding one or more 1-day events to discuss the relevance of model systems in Ayurvedic research, and more specifically the need for organism-specific formulations as pioneered by Priyadarshini *et al*,? May I further propose that such a discussion could be held as an additional session at the 4th World Ayurveda Congress in December this year? Such an initiative would offer the chance to showcase and capitalize on such improvements.

We should “strike the iron while it is hot.”
